# The impact of post-thaw culture time on clinical outcomes of embryos at different developmental stages

**DOI:** 10.3389/fphys.2026.1929566

**Published:** 2026-10-30

**Authors:** Mingting Jiang, Yilu Zou, Zhiren Liu

**Affiliations:** 1 The School of Basic Medical Sciences, Fujian Medical University, Fujian, Fuzhou, China; 2 Center for Reproductive Medicine, The First Affiliated Hospital of Fujian Medical University, Fujian, Fuzhou, China

**Keywords:** blastocyst, cleavage-stage embryo, frozen embryo transfer, live birth, post-thaw culture time

## Abstract

**Purpose:**

This study aims to investigate the effect of post-thaw embryo culture time on the clinical outcomes of embryos at different developmental stages.

**Methods:**

This study adopted a retrospective analysis design, in which frozen embryo transfer cycles were divided into four subgroups: single cleavage-stage embryo transfer cycles, double cleavage-stage embryo transfer cycles, single blastocyst transfer cycles, and double blastocyst transfer cycles. Univariate and multivariate analyses were performed to evaluate the effect of post-thaw culture time on live birth rate, gestational age at birth, birth weight, and body length at birth within each subgroup. The live birth rate was defined as the primary outcome.

**Results:**

In double cleavage-stage embryo transfer cycles (n=936), the <4h post-thaw embryo culture group had a significantly lower live birth rate (45.1%) than the ≥4h group (57.2%), with *P* = 0.001. After adjusting for confounding factors in the multivariable regression model, post-thaw culture time remained significantly associated with live birth rate (*P* = 0.008), and the adjusted OR (95% CI) was 1.524 (1.117–2.081). In single blastocyst transfer cycles (n=688), the <4h post-thaw embryo culture group had a significantly higher live birth rate (47.2%) compared with the ≥4h group (34.3%), with *P* = 0.017. After adjusting for confounding factors, post-thaw culture time still maintained a significant association with live birth rate (*P* = 0.003), and the adjusted OR (95% CI) was 0.493 (0.308–0.788). A statistically significant interaction was detected (adjusted interaction OR = 0.424, 95%CI 0.272–0.663, P<0.001), confirming that the effect of post-thaw culture time on live birth rate differs between cleavage-stage and blastocyst-stage embryos.

**Conclusion:**

Post-thaw culture time significantly impacts live birth rates in both double cleavage-stage embryo transfer cycles and single blastocyst transfer cycles, and the effect varies according to the embryonic developmental stage.

## Introduction

The development and implementation of vitrification have had a profound impact on assisted reproductive technology (ART). Embryo vitrification technology has the potential to significantly enhance embryo survival rates, thereby offering substantial benefits in terms of live birth rates and safety within the field of ART. Consequently, as this technology becomes more widely adopted, there is an increasing acceptance towards frozen embryo transfer (FET) and single embryo transfer (Nagy et al., 2020).

In a multicenter trial, the authors observed that frozen cleavage-stage embryo transfer in infertile women with polycystic ovary syndrome was associated with a higher live birth rate and a reduced risk of ovarian hyperstimulation syndrome compared to fresh cleavage-stage embryo transfer (Chen et al., 2016). In a subsequent multicenter, randomized trial, the authors observed no significant difference in live-birth rates between fresh cleavage-stage embryo transfer and frozen cleavage-stage embryo transfer among ovulatory women with infertility; however, frozen cleavage-stage embryo transfer was associated with a decreased risk of ovarian hyperstimulation syndrome (Shi et al., 2018). Additionally, an alternative multicenter trial revealed that frozen single blastocyst transfer resulted in higher rates of singleton live birth when compared to fresh single blastocyst transfer (Wei et al., 2019).

The results of all these multicenter trials demonstrate the evident advantages of frozen embryo transfer in various aspects. Therefore, optimizing the procedures associated with frozen embryo transfer holds significant clinical value.

The optimization of the duration of culture for frozen cleavage-stage embryos from thawing to transfer is a focused procedure that remains controversial. A randomized controlled trial demonstrated that transferring frozen cleavage-stage embryos 18–24 h after thaw did not significantly increase ongoing pregnancy rates compared to transfer within 2–5 h after thaw (Colodetti et al., 2020). Multiple independent studies consistently reported that prolonged culture following thawing of cleavage-stage embryos yielded equivalent clinical pregnancy rates to short-duration culture (Friedman et al., 2022; Ranji et al., 2023; Wang et al., 2023). However, some studies suggested that extending the culture time of thawed cleavage-stage embryos until blastocyst stage before transfer was a beneficial strategy (Li et al., 2021; Aytac and Kilicdag, 2022; Madani et al., 2024). Therefore, the optimal culture time for thawed cleavage-stage embryos continues to be debated.

The optimal post-thaw culture time for blastocysts remains a subject of controversy too. While previous study showed that the length of culture time after thawing did not impact the success rate of FET cycles (Pham et al., 2022), other study suggested that extending the interval between thawing and transfer might enhance the selection process for frozen-thawed blastocysts (Guerif et al., 2003).

In summary, the optimal post-thaw culture time for both cleavage stage and blastocyst stage embryos remains a contentious issue, and this study aims to address these unresolved questions.

## Materials and methods

### Criteria for patient selection

The present study provides a retrospective analysis of FET cycles conducted at our institution from September 2018 to October 2023. The inclusion criteria for the analyzed cycles were defined as follows (1): The FET cycle was performed using cleavage-stage embryos, which were categorized as grade 1-2 (2); The FET cycle was conducted using blastocysts graded ≥3BB (3); This FET cycle was the patient’s first embryo transfer performed at our center (4). For cycles involving double embryo transfer, both embryos were thawed and transferred simultaneously following the same culture interval, and both embryos were required to meet pre-specified quality criteria.

The exclusion criteria were as follows: (1) Patients with acquired or congenital uterine malformations; (2) Thawed cleavage-stage embryos were cultured to the blastocyst stage before transfer; (3) A subsequent frozen-thawed embryo transfer cycle was performed. A representative protocol involves the transfer of one cleavage-stage embryo on day 3, followed by the transfer of a blastocyst on day 5. (4) At least one thawed cleavage-stage embryo failed to achieve a grade of 1–2; (5) Morphological assessment of thawed cleavage-stage embryos via inverted microscopy revealed that one or more blastomeres displayed morphological features characteristic of apoptosis, including cell lysis and abnormal cellular shrinkage; (6) At least one thawed blastocyst presented with a grade C inner cell mass (ICM) or trophectoderm (TE) score; (7) Following thawing, the blastocyst failed to re-expand, and morphological assessment revealed that over 50% of its cells exhibited morphological characteristics consistent with apoptosis. Since our institution has not yet implemented preimplantation genetic testing (PGT) technology, there is no need to specifically exclude FET cycles involving PGT. The inclusion and exclusion criteria for the FET cycles and the specific grouping protocols of this study are presented in **Figure 1**.

**Figure 1:**
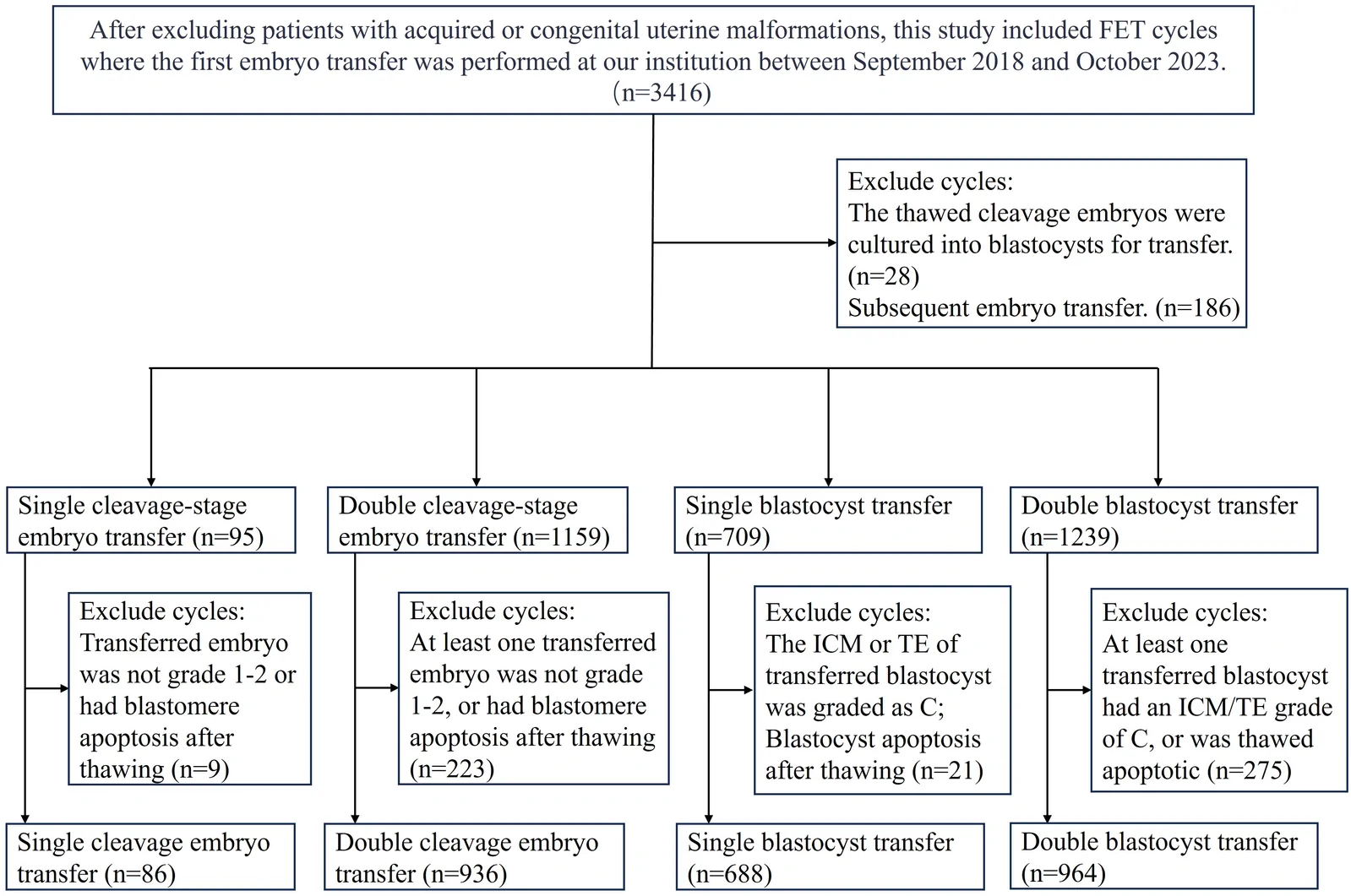
Schematic diagram illustrating the filtration of FET cycles.

### Ovarian stimulation and *in vitro* fertilization

Ovarian stimulation protocols, including the GnRH-agonist prolonged protocol, GnRH-agonist long protocol, GnRH-antagonist protocol, mini-stimulation protocol, luteal phase ovarian stimulation protocol, and natural cycle, were utilized to treat the patients included in the study. In protocols that required the use of gonadotrophin (Gn), the dosage of Gn was adjusted according to follicular development as indicated by ultrasound monitoring and serum estradiol levels. When ultrasound monitoring confirmed that at least one follicle reached a diameter of 17 mm, human chorionic gonadotropin (hCG) was administered to induce the final maturation of the oocytes. Approximately 36 h later, transvaginal ultrasound-guided oocyte retrieval was conducted to obtain the cumulus-oocyte complexes. These cumulus-oocyte complexes were then transferred to the embryo laboratory for *in vitro* fertilization (IVF).

IVF was typically categorized into two primary methods: conventional IVF and intracytoplasmic sperm injection (ICSI). The selection of the IVF method was primarily influenced by the quality of the male partner’s sperm. Because PGT had not yet been approved for use at our center during the implementation of this study, no patients were chosen for ICSI specifically for PGT purposes.

### Embryo culture and score

The embryos were cultured in a sequential medium and incubated at 37 °C under a gas mixture composed of 6% CO_2_, 5% O_2_, and 89% N_2_, with the culture being covered by oil. The main reagents employed for culture included IVF-PLUS, G1-PLUS, G2-PLUS, EmbryoGlue, and Ovoil (Vitrolife Sweden AB, Sweden). These reagents remained constant throughout the study duration.

Assessment of thawed embryos was performed at two distinct stages: immediate evaluation post-thawing and pre-transfer evaluation. In this study, we observed no transfer cycles in which embryos remained intact at the immediate post-thaw assessment, but developed apoptotic blastomeres prior to transfer. The assessment of cleavage-stage embryos and the definition of grade 1–2 embryos were based on the previous Istanbul consensus workshop (Alpha Scientists in Reproductive Medicine ESIGoE, 2011). The evaluation of cleavage-stage embryo grades involved considering factors such as cell number, percentage of fragmentation, uniformity in cell size, and the presence of multinucleated or vacuolated blastomeres. Blastocysts were evaluated based on Gardner and Schoolcraft’s classification system (Gardner et al., 2000), which assesses the quantity and integrity of both ICM and TE cells, as well as the expansion of the blastocoel cavity. The panel of embryologists responsible for evaluating the embryos remained constant throughout the study and successfully passed the evaluation conformance test. The performance of each embryologist was subject to regular internal quality control measures as well as external quality assessments.

### Embryo vitrification, thawing, post-thaw culture, and endometrial preparation

Cleavage-stage embryo cryopreservation was typically performed on day 3, when embryos contained 7–9 blastomeres and were graded as quality 1 or 2. Blastocyst-stage embryos were conventionally cryopreserved after reaching the blastocyst stage. All day 5 blastocysts, except those allocated for fresh embryo transfer, were subjected to cryopreservation. Any remaining embryos that developed into blastocysts by day 6 were also cryopreserved. As a result, day 5 blastocysts accounted for a relatively higher proportion of the total frozen blastocyst cohort.

A vitrification kit (Kitazato, BioPharma, Shizuoka, Japan) was used for embryo vitrification. The specific procedure involved transferring the embryo from the culture medium to the equilibration solution, and assessing its equilibrium state by observing changes in embryo size. Blastomeres were considered to have reached equilibrium when they recovered to over 80% of their initial volume. Following equilibration, the embryo was transferred to the vitrification solution and promptly placed on a Cryotop (Kitazato, BioPharma, Shizuoka, Japan) after 1 minute. Subsequently, the Cryotop was immersed in liquid nitrogen for complete vitrification.

When thawing the embryo, locate the corresponding Cryotop based on its location information. Subsequently, expedite the transfer of the Cryotop containing the embryos from liquid nitrogen to a preheated thawing solution at 37 °C. After a duration of 1 minute, proceed with transferring the embryos to a dilution solution for 3 minutes, followed by wash solution 1 for 5 minutes and wash solution 2 for 1 minute. Finally, transfer the embryos into G2-PLUS culture medium and incubate them at 37 °C in a humidified atmosphere containing 6% CO_2_, 5% O_2_, and 89% N_2_, with the culture dishes overlaid with mineral oil.

The post-thaw culture time of embryos was defined as the interval from the completion of the thawing procedure and transfer of embryos into G2 culture medium to the final transfer of embryos into the maternal uterus. Embryo thawing was routinely scheduled between 8:30 a.m. and 9:30 a.m. on the day of embryo transfer, and the specific timing of the transfer procedure was determined via joint consultation between the clinical medical team and the patient. To minimize selection bias associated with work scheduling to the greatest extent possible, we compared baseline characteristics between the two groups defined by post-thaw culture time (< 4 h vs. ≥ 4 h), and incorporated covariates with statistically significant intergroup differences into the subsequent multivariate regression model. Baseline characteristic comparisons are presented in **Supplementary Tables 1**-**4**.

The natural cycle and exogenous steroid replacement cycle were utilized for endometrial preparation. In the natural cycle, once the dominant follicle reached a diameter of 16–18 mm as monitored by ultrasound, ovulation was triggered with an hCG injection, followed by the initiation of progesterone administration 36 h post-injection. In the exogenous steroid replacement cycle, estradiol valerate tablets were administered orally at a dose of 4–6 mg daily, commencing on the third day of the menstrual cycle. Ultrasound monitoring of the endometrial lining began on the eighth day of the cycle, and the dosage was adjusted as needed based on the results. When the endometrial thickness exceeded 7 mm, progesterone was administered via intramuscular injection or dydrogesterone tablets were taken orally. Prior to initiating estradiol valerate treatment, Triptorelin Acetate for Injection was administered to achieve pituitary suppression, depending on the patient’s specific condition.

### Classification of endometrial morphology and definitions of selected variables

Pattern A endometrium was hyperechogenic relative to the myometrium, with the central echogenic line undetectable (Gonen and Casper, 1990). Pattern B was an intermediate endometrial pattern that was isoechogenic compared to the myometrium, in which the central echogenic line was difficult to visualize (Gonen and Casper, 1990). Pattern C was a multilayered endometrial pattern (also designated the “triple-line” pattern), consisting of a distinct midline hyperechogenic line and outer hyperechogenic lines, with hypoechoic or anechoic regions between the lines that correspond to the functional layer of the endometrium (Gonen and Casper, 1990). Transitional mixed categories A-B and B-C were additionally assigned to cycles that exhibited intermediate ultrasonic features corresponding to A-B and B-C, respectively.

Classification of male infertility: primary infertility was defined as the male partner having no prior biological children; secondary infertility was defined as the male partner having previously had biological offspring. Duration of male infertility referred to the total number of years during which a man has attempted to achieve pregnancy with his partner(s) — including both previous and current partners — without successfully conceiving.

### Statistical analysis

When assessing the potential impact of post-thaw culture time on live birth outcomes, the included cycles were categorized into “No live birth” and “Live birth” groups, followed by univariate analysis. Student’s t-test was applied for normally distributed continuous variables; Welch’s corrected t-test was used for normally distributed data with unequal variances. The Mann-Whitney U test was used for continuous variables with non-normal distributions. Categorical variables were examined using the chi-square test. Subsequently, multivariate analysis (binary logistic regression) was performed to adjust for variables that had a *P*-value < 0.10 in the univariate analysis, as well as those showed significant between-group differences at baseline after stratification by post-thaw culture time.

Live birth was defined as the delivery of one or more infants showing signs of life after at least 24 completed weeks of gestation. Perinatal outcomes were collected via the hospital electronic medical record system and telephone follow-up. No participants were lost to follow-up throughout the course of this study. The primary outcome was binary: live birth (yes/no). The negative group (no live birth) included implantation failure, biochemical pregnancy, clinical miscarriage, ectopic pregnancy, stillbirth, and pregnancy termination.

When evaluating the potential impact of post-thaw culture time on gestational age at birth, birth weight, and body length at birth, the post-thaw culture time was analyzed as a continuous variable. univariate analysis was conducted using simple linear regression, while multivariate analysis employed multiple linear regression. Confounding variables were defined as those with a *P*-value less than 0.1 in the simple linear regression analysis.

## Results

The minimum duration of culture time following embryo thawing in the included cycles was 0.1 h, while the maximum was 8.7 h, with a mean ± standard deviation of 3.1 ± 1.9 h. The frequency distribution for each time period is depicted in **Figure 2**. It can be observed from **Figure 2** that the distribution of culture time following embryo thawing exhibits two distinct segments approximately separated by a threshold of 4 h. Therefore, for our statistical analysis on live birth outcomes, we categorized the culture time following embryo thawing into two groups based on this threshold: the <4h group and the ≥4h group.

**Figure 2:**
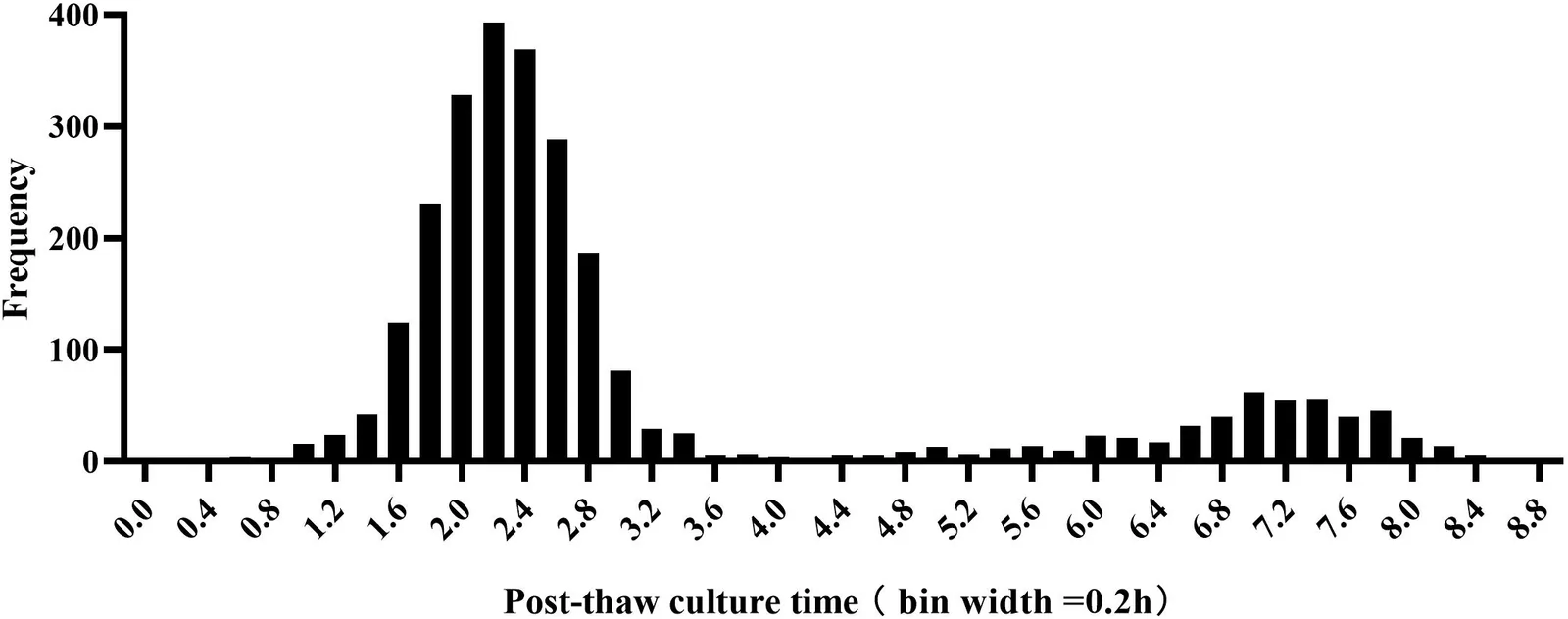
The distribution of culture time following embryo thawing. The unit of time is h. Bin width=0.2 h.

To more accurately analyze the impact of culture time following embryo thawing on outcomes, we categorized the included FET cycles into four groups: single cleavage-stage embryo transfer, double cleavage-stage embryo transfer, single blastocyst transfer, and double blastocyst transfer. The frequency distribution of post-thaw culture time across these four subgroups is presented in **Supplementary Figure S1**.

In single cleavage-stage embryo transfer cycles (**Table 1**), univariate analysis revealed that both female and male age significantly influenced live birth outcomes, while the post-thaw culture time did not have a discernible impact. However, there was a slightly higher live birth rate observed in the ≥4h group (30%) compared to the <4h group (25%). Additionally, **Supplementary Table 1** presented significant between-group differences in female infertility duration and female BMI when baseline characteristics were compared across post-thaw culture time groups. These two variables were incorporated into a multivariable binary logistic regression model, along with variables that yielded *P* < 0.1 in univariate analysis. The results indicated that after adjusting for female age, female infertility duration, female BMI and male age, post-thaw culture time had no significant association with live birth outcomes.

**Table 1:** Univariate and multivariate analyses were conducted to assess the impact of post-thaw culture time on live birth outcomes in single cleavage embryo transfer cycles.

	No live birth 63 (73.3%)	Live birth 23 (26.7%)	*P*-value from univariate analysis	P-value and OR (95% CI) from multivariate analysis d
Post-thaw culture time (n, %)				
< 4 h	42 (75.0%)	14 (25.0%)		Ref
≥ 4 h	21 (70.0%)	9 (30.0%)	0.618^a^	0.860^f^, 1.107(0.358, 3.421)
Female age	36.7±6.7	32.0±4.3	<0.001^b^	0.441^e^, 0.942(0.808, 1.097)
Female infertility type (n, %)				
primary infertility	28 (70.0%)	12 (30.0%)		
secondary infertility	35 (76.1%)	11 (23.9%)	0.525^a^	
Female infertility duration (years)	3.3 (3.9)	3.4 (4.0)	0.819^c^	0.723^e^, 0.967(0.804, 1.163)
Female BMI	22.3±3.4	21.8±2.6	0.519^b^	0.793^e^, 0.979(0.834, 1.149)
Endometrial thickness (mm)	9.4±1.7	9.7±2.2	0.501^b^	
Male age	38.3±6.6	33.6±4.2	<0.001^b^	0.285^e^, 0.923(0.797, 1.069)
Male infertility type (n, %)				
primary infertility	34 (69.4%)	15 (30.6%)		
secondary infertility	29 (78.4%)	8 (21.6%)	0.351^a^	
Male infertility duration (years)	2.1 (4.6)	2.1 (5.5)	0.444^c^	
Male BMI	24.3±3.2	23.5±3.5	0.349^b^	
Endometrial preparation protocol (n, %)				
natural cycle	14 (77.8%)	4 (22.2%)		
exogenous steroid replacement cycle	49 (72.1%)	19 (27.9%)	0.626^a^	
Endometrial morphology (n, %)				
A	1 (100.0%)	0 (0.0%)		
A–B	4 (80.0%)	1 (20.0%)		
B	4 (57.1%)	3 (42.9%)		
B-C	7 (63.6%)	4 (36.4%)		
C	47 (75.8%)	15 (24.2%)	0.711^a^	

^a^ Pearson χ^2^ test. Values are number (percentage).

^b^ Two-sample t-test. Values are mean ± SD.

^c^ Two-sample Mann–Whitney test. Values are median (IQR).

^d^ Multivariate analysis (binary logistic regression) was performed to adjust for variables that had a *P*-value < 0.10 in the univariate analysis, as well as those that showed significant between-group differences at baseline after stratification by post-thaw culture time.

^e^ *P*-value of each variable’s overall effects after adjusting for the other variables.

^f^ *P*-value between each variable’s subgroups and reference group.

In double cleavage-stage embryo transfer cycles (**Table 2**), the univariate analysis revealed a significantly higher live birth rate in the ≥4h group (57.2%) compared to the <4h group (45.1%). Confounding variables, including female age, female infertility type, female infertility duration, endometrial thickness, male age, male infertility type, male infertility duration, endometrial preparation protocol, and endometrial morphology, were identified based on two sources: the results of univariate analysis, and the significant between-group differences at baseline after stratification by post-thaw culture time presented in **Supplementary Table 2**. After adjusting for these confounding variables, multivariate analysis confirmed that post-thaw culture time had a significant impact on live birth.

**Table 2:** Univariate and multivariate analyses were conducted to assess the impact of post-thaw culture time on live birth outcomes in double cleavage embryo transfer cycles.

	No live birth 477 (51.0%)	Live birth 459 (49.0%)	*P*-value from univariate analysis	*P*-value and OR (95% CI) from multivariate analysis ^d^
Post-thaw culture time (n, %)				
< 4 h	346 (54.9%)	284 (45.1%)		Ref
≥ 4 h	131 (42.8%)	175 (57.2%)	0.001^a^	0.008^f^, 1.524(1.117, 2.081)
Female age	34.7±5.7	30.8±4.1	<0.001^b^	<0.001^e^, 0.894(0.852, 0.938)
Female infertility type (n, %)				
primary infertility	254 (45.6%)	303 (54.4%)		Ref
secondary infertility	223 (58.8%)	156 (41.2%)	<0.001^a^	0.916^f^, 1.018(0.732, 1.416)
Female infertility duration (years)	3.9 (4.1)	3.2 (2.8)	<0.001^c^	0.021^e^, 0.932(0.878, 0.989)
Female BMI	22.2±3.2	22.0±3.3	0.359^b^	
Endometrial thickness (mm)	9.3±1.9	9.8±1.9	<0.001^b^	0.005^e^, 1.115(1.034, 1.203)
Male age	36.2±6.2	32.3±4.5	<0.001^b^	0.110^e^, 0.964(0.922, 1.008)
Male infertility type (n, %)				
primary infertility	303 (48.0%)	328 (52.0%)		Ref
secondary infertility	174 (57.1%)	131 (43.0%)	0.010^a^	0.596^f^, 0.914(0.655, 1.275)
Male infertility duration (years)	1.7 (4.5)	1.7 (3.1)	0.054^c^	0.934^e^, 1.002(0.950, 1.057)
Male BMI	24.5±3.6	24.7±3.6	0.333^b^	
Endometrial preparation protocol (n, %)				
natural cycle	79 (67.5%)	38 (32.5%)		Ref
exogenous steroid replacement cycle	398 (48.6%)	421 (51.4%)	<0.001^a^	0.083^f^, 1.498(0.949, 2.364)
Endometrial morphology (n, %)				
A	7 (53.8%)	6 (46.2%)		0.900^f^, 0.926(0.280, 3.066)
A–B	61 (43.6%)	79 (56.4%)		0.621^f^, 1.112(0.731, 1.691)
B	45 (51.7%)	42 (48.3%)		0.731^f^, 0.917(0.560, 1.501)
B–C	54 (50.9%)	52 (49.1%)		0.372^f^, 0.812(0.513, 1.283)
C	310 (52.5%)	280 (47.5%)	0.446^a^	Ref

^a^ Pearson χ^2^ test. Values are number (percentage).

^b^ Two-sample t-test. Values are mean ± SD.

^c^ Two-sample Mann–Whitney test. Values are median (IQR).

^d^ Multivariate analysis (binary logistic regression) was performed to adjust for variables that had a *P*-value < 0.10 in the univariate analysis, as well as those that showed significant between-group differences at baseline after stratification by post-thaw culture time.

^e^ *P*-value of each variable’s overall effects after adjusting for the other variables.

^f^ *P*-value between each variable’s subgroups and reference group.

In single blastocyst transfer cycles (**Table 3**), the univariate analysis revealed a significantly higher live birth rate in the <4h group (47.2%) compared to the ≥4h group (34.3%). Confounding variables included in the multivariable regression model were selected based on the results of univariate analysis and baseline characteristic comparisons presented in **Supplementary Table 3**. After adjusting for the confounding variables, including female age, female infertility type, female infertility duration, female BMI, endometrial thickness, male age, male infertility type, and male infertility duration, multivariate analysis confirmed that post-thaw culture time had a significant impact on live birth.

**Table 3:** Univariate and multivariate analyses were conducted to assess the impact of post-thaw culture time on live birth outcomes in single blastocyst transfer cycles.

	No live birth 376 (54.7%)	Live birth 312 (45.3%)	*P*-value from univariate analysis	*P*-value and OR (95% CI) from multivariate analysis ^d^
Post-thaw culture time (n, %)				
< 4 h	311 (52.8%)	278 (47.2%)		Ref
≥ 4 h	65 (65.7%)	34 (34.3%)	0.017^a^	0.003^f^, 0.493(0.308, 0.788)
Female age	32.7±5.0	30.9±4.2	<0.001^b^	0.120^e^, 0.955(0.900, 1.012)
Female infertility type (n, %)				
primary infertility	199 (49.9%)	200 (50.1%)		Ref
secondary infertility	177 (61.2%)	112 (38.8%)	0.003^a^	0.407^f^, 0.858(0.598, 1.232)
Female infertility duration (years)	3.8 (3.8)	3.4 (2.9)	0.002^c^	0.554^e^, 0.980(0.916, 1.048)
Female BMI	22.1±3.3	21.3±3.1	<0.001^b^	0.007^e^, 0.932(0.885, 0.981)
Endometrial thickness (mm)	9.0±2.1	9.7±2.1	<0.001^b^	<0.001^e^, 1.182(1.093, 1.279)
Male age	34.7±5.4	32.7±4.6	<0.001^b^	0.462^e^, 0.980(0.930, 1.033)
Male infertility type (n, %)				
primary infertility	205 (50.5%)	201 (49.5%)		Ref
secondary infertility	171 (60.6%)	111 (39.4%)	0.009^a^	0.267^f^, 0.818(0.574, 1.166)
Male infertility duration (years)	2.2 (4.0)	1.7 (4.0)	0.069^c^	0.602^e^, 0.984(0.925, 1.046)
Male BMI	24.2±3.3	24.3±3.5	0.723^b^	
Endometrial preparation protocol (n, %)				
natural cycle	73 (60.8%)	47 (39.2%)		
exogenous steroid replacement cycle	303 (53.3%)	265 (46.7%)	0.134^a^	
Endometrial morphology (n, %)				
A	0 (0.0%)	1 (100.0%)		
A–B	17 (42.5%)	23 (57.5%)		
B	20 (57.1%)	15 (42.9%)		
B–C	20 (51.3%)	19 (48.7%)		
C	319 (55.7%)	254 (44.3%)	0.393^a^	

^a^ Pearson χ^2^ test. Values are number (percentage).

^b^ Two-sample t-test. Values are mean ± SD.

^c^ Two-sample Mann–Whitney test. Values are median (IQR).

^d^ Multivariate analysis (binary logistic regression) was performed to adjust for variables that had a *P*-value < 0.10 in the univariate analysis, as well as those that showed significant between-group differences at baseline after stratification by post-thaw culture time.

^e^ *P*-value of each variable’s overall effects after adjusting for the other variables.

^f^ *P*-value between each variable’s subgroups and reference group.

In double blastocyst transfer cycles (**Table 4**), although the <4h group (55.2%) exhibited a slightly higher live birth rate compared to the ≥4h group (46.1%), no statistically significant difference was observed. Confounding variables included in the multivariable regression model were selected based on the results of univariate analysis and baseline characteristic comparisons presented in **Supplementary Table 4**. Upon adjusting for the confounding variables, including female age, female infertility type, female infertility duration, endometrial thickness, male age, male infertility type, male infertility duration, and endometrial preparation protocol, multivariate analysis confirmed that post-thaw culture time had no significant impact on live birth outcomes.

**Table 4:** Univariate and multivariate analyses were conducted to assess the impact of post-thaw culture time on live birth outcomes in double blastocyst transfer cycles.

	No live birth 439 (45.5%)	Live birth 525 (54.5%)	*P*-value from univariate analysis	*P*-value and OR (95% CI) from multivariate analysis ^d^
Post-thaw culture time (n, %)				
< 4 h	398 (44.8%)	490 (55.2%)		Ref
≥ 4 h	41 (53.9%)	35 (46.1%)	0.125^a^	0.479^f^, 0.834(0.505, 1.378)
Female age	33.7±4.7	31.1±4.1	<0.001^b^	<0.001^e^, 0.877(0.836, 0.920)
Female infertility type (n, %)				
primary infertility	208 (41.0%)	299 (59.0%)		Ref
secondary infertility	231 (50.5%)	226 (49.5%)	0.003^a^	0.190^f^, 0.798(0.569, 1.119)
Female infertility duration (years)	4.3 (4.0)	3.7 (3.0)	<0.001^c^	0.169^e^, 0.965(0.916, 1.015)
Female BMI	22.2±3.1	22.0±3.4	0.278^b^	
Endometrial thickness (mm)	8.9±1.9	9.2±1.8	0.009^b^	0.036^e^, 1.082(1.005, 1.165)
Male age	35.4±5.7	33.1±5.0	<0.001^b^	0.639^e^, 1.009(0.971, 1.050)
Male infertility type (n, %)				
primary infertility	263 (43.3%)	344 (56.7%)		Ref
secondary infertility	176 (49.3%)	181 (50.7%)	0.072^a^	0.290^f^, 1.208(0.852, 1.712)
Male infertility duration (years)	2.0 (4.0)	2.0 (3.0)	0.032^c^	0.409^e^, 0.979(0.931, 1.029)
Male BMI	24.2±3.3	24.3±3.7	0.722^b^	
Endometrial preparation protocol (n, %)				
natural cycle	119 (56.9%)	90 (43.1%)		Ref
exogenous steroid replacement cycle	320 (42.4%)	435 (57.6%)	<0.001^a^	<0.001 ^f^, 1.794(1.296, 2.484)
Endometrial morphology (n, %)				
A	0 (0.0%)	1 (100.0%)		
A–	14 (38.9%)	22 (61.1%)		
B	10 (31.3%)	22 (68.8%)		
B–C	19 (40.4%)	28 (59.6%)		
C	396 (46.7%)	452 (53.3%)	0.280^a^	

^a^ Pearson χ^2^ test. Values are number (percentage).

^b^ Two-sample t-test. Values are mean ± SD.

^c^ Two-sample Mann–Whitney test. Values are median (IQR).

^d^ Multivariate analysis (binary logistic regression) was performed to adjust for variables that had a *P*-value < 0.10 in the univariate analysis, as well as those that showed significant between-group differences at baseline after stratification by post-thaw culture time.

^e^ *P*-value of each variable’s overall effects after adjusting for the other variables.

^f^ *P*-value between each variable’s subgroups and reference group.

The results from the four aforementioned subgroups indicated that the association between post-thaw culture time and live birth rate may vary depending on the developmental stage of the transferred embryos. To validate this hypothesis, we performed a comprehensive multivariate regression analysis by pooling all FET cycles across the four subgroups. Variables with a *P*-value < 0.10 in the univariate analysis, or variables showing significant between-group differences at baseline after stratification by post-thaw culture time, were incorporated into the model. In addition, the model also included post-thaw culture time, embryo developmental stage, number of transferred embryos, and the two-way interaction term between post-thaw culture time and embryo developmental stage.

A statistically significant interaction was detected (adjusted interaction OR = 0.424, 95%CI 0.272–0.663, *P* < 0.001), confirming that the effect of post-thaw culture time on live birth rate differs between cleavage-stage and blastocyst-stage embryos. Stratified analyses presented in **Tables 1**-**4** further characterized the direction and magnitude of the associations within each transfer subgroup. The full output of the pooled regression model is provided in **Supplementary Table 5**.

We further performed exploratory analyses to investigate the potential effects of post-thaw culture time on gestational age at birth, birth weight, and body length at birth; the detailed results are reported in **Supplementary Tables 6**-**9**.

In addition, these secondary reproductive outcomes across four subgroups stratified by post-thaw culture time, such as clinical pregnancy rates, miscarriage rates, and multiple pregnancy rates, are presented in **Supplementary Table 10** to explore at which stage the group-level difference originates.

## Discussion

The prevailing consensus is that the intrauterine embryo culture environment surpasses the *in vitro* culture environment (Sciorio and Rinaudo, 2023), prompting most medical professionals and embryologists to promptly transfer thawed embryos into the intrauterine cavity to facilitate their development.

Conversely, certain medical professionals and embryologists contend that vitrification and thawing of embryos may result in damage to the embryo (Sciorio et al., 2024). Therefore, it is imperative for these embryos to be appropriately cultured in a stable environment post-thawing until they have undergone basic recovery, so as to withstand any further harm caused by the *in vitro* operation during transfer. While some doctors and embryologists have achieved success through extending the duration of post-thaw culture time, there remains insufficient statistical validation with respect to sample size to determine whether this approach applies universally across all types of embryos.

Based on the aforementioned arguments, this study aimed to compare the clinical outcomes between embryo culture times of less than 4 h and more than 4 h after thawing. The objective was to provide evidence for doctors and embryologists in determining the appropriate culture time.

Importantly, the clinical outcomes were compared among four subgroups of FET cycles: single cleavage-stage embryo transfer, double cleavage-stage embryo transfer, single blastocyst transfer, and double blastocyst transfer. This subgroup division was determined based on both the developmental stage of the transferred embryos (cleavage or blastocyst) and the number of transferred embryos, factors that significantly influence FET cycle outcomes (Parsafar and Dehghani-Firouzabadi, 2023; Zhou et al., 2024). This effect is supported by our findings, as shown in **Tables 1**-**4**, which demonstrate differences in live birth rates across the four subgroups of FET cycles.

Furthermore, double embryo transfer is more likely to result in twin live births (Chen et al., 2022), and the TE grade is an independent risk factor for monozygotic twinning following single blastocyst transfer (Ge et al., 2023). Therefore, the number of transferred embryos and their developmental stage can significantly influence the number of live births, which, in turn, impacts clinical outcomes such as gestational age at birth and birth weight, as demonstrated in our **Supplementary Tables 6**-**9**. Consequently, categorizing the included cycles into the aforementioned four subgroups will effectively minimize bias and enhance the accuracy of the results.

### Frozen cleavage-stage embryo transfer

Our study has demonstrated that in double cleavage-stage embryo transfer cycles, a post-thaw culture time of ≥4 h resulted in significantly higher live birth rates compared to those with <4 h. This finding suggests that extending the culture period for two thawed cleavage-stage embryos prior to transfer confers an advantage in enhancing live birth rates within a culture time range of 0.1-8.7 h.

The finding contradicts previous reports (Colodetti et al., 2020; Friedman et al., 2022; Ranji et al., 2023; Wang et al., 2023), potentially due to the variable length of extended cultivation. In our study, the extended culture time ranged from 4 to 8.7 h, whereas previous studies utilized an extended culture time of 18 to 20 h or more. Furthermore, unlike these prior studies (Colodetti et al., 2020; Friedman et al., 2022; Ranji et al., 2023; Wang et al., 2023) that primarily focused on clinical or ongoing pregnancy rates as outcomes, we employed live birth rates as our primary outcome measure.

In addition, several studies have suggested that prolonging the culture duration of thawed cleavage-stage embryos until the blastocyst stage prior to transfer may represent a promising strategy (Li et al., 2021; Aytac and Kilicdag, 2022; Madani et al., 2024). While these studies align with our findings and endorse the extension of post-thaw culture time for cleavage-stage embryos, it is noteworthy that their methodology involves prolonging culture until the blastocyst stage, potentially introducing a bias in assessing the impact of post-thaw culture time on outcomes by excluding embryos incapable of developing into blastocysts.

**Table 1** indicates that in single cleavage-stage embryo transfer cycles, both female age and male age exhibited significant associations with the outcome in univariate analysis, but no significant associations were observed in multivariate analysis. We hypothesized that collinearity existed between female partner age and male partner age. To resolve this issue, we re-performed multivariate analysis after excluding male age from the model. The updated results showed that female age had a significant effect on live birth outcome (*P* = 0.008), with an adjusted odds ratio (95% CI) of 0.882 (0.803, 0.968). The effect of post-thaw culture time on live birth outcome remained non-significant (*P* = 0.891), with an adjusted odds ratio (95% CI) of 1.081 (0.352, 3.323).

### Frozen blastocyst transfer

Our findings suggest that within the investigated range of post-thaw embryo culture time (0.1–8.7 h), single blastocyst transfer cycles exhibited a significantly higher live birth rate in the group with post-thaw culture time <4 h compared with the group with post-thaw culture time ≥4 h. However, our results contradict two previous studies in this regard. Previous studies suggested that the post-thawing culture time of blastocysts was not associated with the live birth rate (Ciaffaglione et al., 2022; Pham et al., 2022), potentially due to variations in cycle screening and grouping methods compared to our study. For instance, we categorized single embryo transfer and double embryo transfer into separate subgroups for statistical analysis in order to better account for the impact of transferred embryo number on the results. Moreover, we excluded low-quality embryos from our study’s transfer cycles to minimize statistical bias arising from differences in embryo quality. The discrepant outcomes observed in another study (Guerif et al., 2003) may primarily stem from variances in freezing techniques used during an earlier period when slow freezing rather than vitrification was employed.

Regarding gestational age at birth, birth weight, and body length at birth, we observed a significant impact of post-thaw culture time solely on gestational age at birth in single cleavage-stage embryo transfer cycles (n=23). However, given the small sample size, further studies with larger cohorts are necessary to validate this preliminary observation.

There are certain limitations in our study. First, this study is inherently limited by its retrospective design, particularly in relation to data collection. Specifically, the following potential confounding parameters were not available for analysis: female age at oocyte retrieval, duration of cryostorage, day of blastocyst vitrification (day 5 vs day 6), specific morphological grade (rather than just a binary pass/fail threshold), post-warming blastocyst re-expansion status, fertilization method (conventional IVF vs ICSI), infertility diagnosis (e.g., PCOS or endometriosis), number of previous transfer attempts, and calendar year of transfer (a relevant covariate for the study period spanning 2020 to 2022), as well as the identity of the transferring clinician and the time of day of embryo transfer. The unavailability of these parameters may introduce bias into the final results. Secondly, the sample size for single cleavage-stage embryo transfer cycles was relatively small, which could potentially impact the statistical analysis. Lastly, it is worth mentioning that there were fewer cycles in the ≥4 h groups for double blastocyst transfer cycles, which might have an influence on the statistical outcomes.

## Conclusion

Our study demonstrates that within the investigated range of post-thaw embryo culture time (0.1–8.7 h), post-thaw culture time is significantly associated with live birth rates in both double cleavage-stage embryo transfer cycles and single blastocyst transfer cycles. Furthermore, the effect of post-thaw culture time on live birth rates differs by embryonic developmental stage. These findings provide clinical guidance for determining the optimal post-thaw culture time for different embryo transfer strategies.

## Data Availability

The data analyzed in this study is subject to the following licenses/restrictions: The datasets used and/or analysed during the current study are available from the corresponding author on reasonable request. Requests to access these datasets should be directed to Zhiren Liu, renzhiyingbing@163.com.

## Supplementary material

Supplementary Figure 1 The frequency distribution of post-thaw culture time across the four subgroups.
