# Supplementary material for: The impact of post-thaw culture time on clinical outcomes of embryos at different developmental stages
Source: Front Physiol. 2026 Oct 30;17:1929566. doi: 10.3389/fphys.2026.1929566 (PMC13759371; doi:10.3389/fphys.2026.1929566)
Supplement: Supplementary file 1 [file Table1.docx]

**Supplementary Table 1 Baseline characteristics table comparing the <4 h and ≥4 h groups in single cleavage-stage embryo transfer cycles**

|  | Post-thaw culture time < 4 h  56 (65.1%) | Post-thaw culture time ≥ 4 h  30 (34.9%) | *P*-value |
| --- | --- | --- | --- |
| Female age | 36.0±6.6 | 34.3±6.0 | 0.232 ^b^ |
| Female infertility type (n, %) |  |  |  |
| primary infertility | 27 (67.5%) | 13 (32.5%) |  |
| secondary infertility | 29 (63.0%) | 17 (37.0%) | 0.665 ^a^ |
| Female infertility duration (years) | 3.7 (4.6) | 2.9 (3.8) | 0.024 ^c^ |
| Female BMI | 21.6±2.7 | 23.3±3.7 | 0.014 ^b^ |
| Endometrial thickness (mm) | 9.3±1.7 | 9.9±2.1 | 0.102^b^ |
| Male age | 37.5±6.4 | 36.3±6.4 | 0.398 ^b^ |
| Male infertility type (n, %) |  |  |  |
| primary infertility | 32 (66.3%) | 17 (34.7%) |  |
| secondary infertility | 24 (64.9%) | 13 (35.1%) | 0.966 ^a^ |
| Male infertility duration (years) | 2.5 (5.6) | 2.0 (4.2) | 0.404 ^c^ |
| Male BMI | 23.9±3.2 | 24.5±3.5 | 0.429 ^b^ |
| Endometrial preparation protocol (n, %) |  |  |  |
| natural cycle | 12 (66.7%) | 6 (33.3%) |  |
| exogenous steroid replacement cycle | 44 (64.7%) | 24 (35.3%) | 0.626 ^a^ |
| Endometrial morphology (n, %) |  |  |  |
| A | 0 (0.0%) | 1 (100.0%) |  |
| A-B | 4 (80.0%) | 1 (20.0%) |  |
| B | 5 (71.4%) | 2 (28.6%) |  |
| B-C | 6 (54.5%) | 5 (45.5%) |  |
| C | 41 (66.1%) | 21 (33.9%) | 0.550 ^a^ |

^a^ Pearson χ² test. Values are number (percentage).

^b^ Two-sample t-test. Values are mean±SD.

^c^ Two-sample Mann–Whitney test. Values are median (IQR).
