# Supplementary material for: The impact of post-thaw culture time on clinical outcomes of embryos at different developmental stages
Source: Front Physiol. 2026 Oct 30;17:1929566. doi: 10.3389/fphys.2026.1929566 (PMC13759371; doi:10.3389/fphys.2026.1929566)
Supplement: Supplementary file 2 [file Table10.docx]

**Supplementary Table 10 A comparative analysis of secondary outcomes across four subgroups**

|  | Post-thaw culture time < 4h | Post-thaw culture time ≥ 4h | *P*-value ^a^ |
| --- | --- | --- | --- |
| Single cleavage-stage embryo transfer, n/n (%) |  |  |  |
| Clinical pregnancy rate | 17/56 (30.4%) | 14/30 (46.7%) | 0.133 |
| Miscarriage rate | 3/17 (17.6%) | 5/14 (35.7%) | 0.253 |
| Live birth rate | 14/56 (25.0%) | 9/30 (30.0%) | 0.618 |
| Multiple pregnancy rate | 0/14 (0%) | 0/9 (0%) | / |
| Double cleavage-stage embryo transfer, n/n (%) |  |  |  |
| Clinical pregnancy rate | 348/630 (55.2%) | 206/306 (67.3%) | <0.001 |
| Miscarriage rate | 55/348 (15.8%) | 28/206 (13.6%) | 0.481 |
| Live birth rate | 284/630 (45.1%) | 175/306 (57.2%) | 0.001 |
| Multiple pregnancy rate | 87/284 (30.6%) | 63/175 (36.0%) | 0.234 |
| Single blastocyst transfer, n/n (%) |  |  |  |
| Clinical pregnancy rate | 332/589 (56.4%) | 43/99 (43.4%) | 0.017 |
| Miscarriage rate | 50/332 (15.1%) | 8/43 (18.6%) | 0.545 |
| Live birth rate | 278/589 (47.2%) | 34/99 (34.3%) | 0.017 |
| Multiple pregnancy rate | 6/278 (2.2%) | 1/34 (2.9%) | 0.771 |
| Double blastocyst transfer, n/n (%) |  |  |  |
| Clinical pregnancy rate | 573/888 (64.5%) | 44/76 (57.9%) | 0.248 |
| Miscarriage rate | 78/573 (13.6%) | 9/44 (20.5%) | 0.209 |
| Live birth rate | 490/888 (55.2%) | 35/76 (46.1%) | 0.125 |
| Multiple pregnancy rate | 175/490 (35.7%) | 14/35 (40.0%) | 0.840 |

^a^ Pearson χ² test. Values are number / number (percentage).

Clinical pregnancy rate = Number of clinical pregnancy cycles / Number of FET cycles.

Miscarriage rate = Number of miscarriage cycles / Number of clinical pregnancy cycles.

Live birth rate = Number of live birth cycles / Number of FET cycles.

Multiple pregnancy rate = Number of multiple pregnancy cycles / Number of live birth cycles.
