# Supplementary material for: The impact of post-thaw culture time on clinical outcomes of embryos at different developmental stages
Source: Front Physiol. 2026 Oct 30;17:1929566. doi: 10.3389/fphys.2026.1929566 (PMC13759371; doi:10.3389/fphys.2026.1929566)
Supplement: Supplementary file 3 [file Table2.docx]

**Supplementary Table 2 Baseline characteristics table comparing the <4 h and ≥4 h groups in double cleavage-stage embryo transfer cycles**

|  | Post-thaw culture time < 4 h  630 (67.3%) | Post-thaw culture time ≥ 4 h  306 (32.7%) | *P*-value |
| --- | --- | --- | --- |
| Female age | 32.9±5.5 | 32.5±5.1 | 0.234 ^b^ |
| Female infertility type (n, %) |  |  |  |
| primary infertility | 361 (64.8%) | 196 (35.2%) |  |
| secondary infertility | 269 (71.0%) | 110 (29.0%) | 0.048 ^a^ |
| Female infertility duration (years) | 3.4 (3.4) | 3.3 (3.4) | 0.122 ^c^ |
| Female BMI | 22.1±3.3 | 22.1±3.1 | 0.748 ^b^ |
| Endometrial thickness (mm) | 9.4±1.9 | 9.8±2.0 | 0.008 ^b^ |
| Male age | 34.4±6.0 | 34.1±5.1 | 0.412 ^b^ |
| Male infertility type (n, %) |  |  |  |
| primary infertility | 404 (64.0%) | 227 (36.0%) |  |
| secondary infertility | 226 (74.1%) | 79 (25.9%) | 0.002 ^a^ |
| Male infertility duration (years) | 1.8 (3.7) | 1.3 (3.8) | 0.169 ^c^ |
| Male BMI | 24.5±3.7 | 24.7±3.6 | 0.302 ^b^ |
| Endometrial preparation protocol (n, %) |  |  |  |
| natural cycle | 93 (79.5%) | 24 (20.5%) |  |
| exogenous steroid replacement cycle | 537 (65.6%) | 282 (34.4%) | 0.003 ^a^ |
| Endometrial morphology (n, %) |  |  |  |
| A | 11 (84.6%) | 2 (15.4%) |  |
| A-B | 119 (85.0%) | 21 (15.0%) |  |
| B | 43 (49.4%) | 44 (50.6%) |  |
| B-C | 83 (78.3%) | 23 (21.7%) |  |
| C | 374 (63.4%) | 216 (36.6%) | <0.001 ^a^ |

^a^ Pearson χ² test. Values are number (percentage).

^b^ Two-sample t-test. Values are mean±SD.

^c^ Two-sample Mann–Whitney test. Values are median (IQR).
