# Supplementary material for: The impact of post-thaw culture time on clinical outcomes of embryos at different developmental stages
Source: Front Physiol. 2026 Oct 30;17:1929566. doi: 10.3389/fphys.2026.1929566 (PMC13759371; doi:10.3389/fphys.2026.1929566)
Supplement: Supplementary file 4 [file Table3.docx]

**Supplementary Table 3 Baseline characteristics table comparing the <4 h and ≥4 h groups in single blastocyst transfer cycles**

|  | Post-thaw culture time < 4 h  589 (85.6%) | Post-thaw culture time ≥ 4 h  99 (14.4%) | *P*-value |
| --- | --- | --- | --- |
| Female age | 31.9±4.7 | 31.9±5.0 | 0.949 ^b^ |
| Female infertility type (n, %) |  |  |  |
| primary infertility | 339 (85.0%) | 60 (15.0%) |  |
| secondary infertility | 250 (86.5%) | 39 (13.5%) | 0.569 ^a^ |
| Female infertility duration (years) | 3.6 (3.3) | 3.8 (3.2) | 0.548 ^c^ |
| Female BMI | 21.7±3.2 | 21.7±3.2 | 0.856 ^b^ |
| Endometrial thickness (mm) | 9.3±2.1 | 9.7±2.1 | 0.032 ^b^ |
| Male age | 33.8±5.2 | 33.7±5.2 | 0.815 ^b^ |
| Male infertility type (n, %) |  |  |  |
| primary infertility | 342 (84.2%) | 64 (15.8%) |  |
| secondary infertility | 247 (87.6%) | 35 (12.4%) | 0.218 ^a^ |
| Male infertility duration (years) | 2.0 (4.0) | 2.3 (4.0) | 0.480 ^c^ |
| Male BMI | 24.2±3.4 | 24.4±3.4 | 0.649 ^b^ |
| Endometrial preparation protocol (n, %) |  |  |  |
| natural cycle | 99 (82.5%) | 21 (17.5%) |  |
| exogenous steroid replacement cycle | 490 (86.3%) | 78 (13.7%) | 0.285 ^a^ |
| Endometrial morphology (n, %) |  |  |  |
| A | 1 (100.0%) | 0 (0.0%) |  |
| A-B | 35 (87.5%) | 5 (12.5%) |  |
| B | 25 (71.4%) | 10 (28.6%) |  |
| B-C | 31 (79.5%) | 8 (20.5%) |  |
| C | 497 (86.7%) | 76 (13.3%) | 0.100 ^a^ |

^a^ Pearson χ² test. Values are number (percentage).

^b^ Two-sample t-test. Values are mean±SD.

^c^ Two-sample Mann–Whitney test. Values are median (IQR).
