# Supplementary material for: The impact of post-thaw culture time on clinical outcomes of embryos at different developmental stages
Source: Front Physiol. 2026 Oct 30;17:1929566. doi: 10.3389/fphys.2026.1929566 (PMC13759371; doi:10.3389/fphys.2026.1929566)
Supplement: Supplementary file 5 [file Table4.docx]

**Supplementary Table 4 Baseline characteristics table comparing the <4 h and ≥4 h groups in double blastocyst transfer cycles**

|  | Post-thaw culture time < 4 h  888 (92.1%) | Post-thaw culture time ≥ 4 h  76 (7.9%) | *P*-value |
| --- | --- | --- | --- |
| Female age | 32.2±4.6 | 33.2±4.6 | 0.058 ^b^ |
| Female infertility type (n, %) |  |  |  |
| primary infertility | 472 (93.1%) | 35 (6.9%) |  |
| secondary infertility | 416 (91.0%) | 41 (9.0%) | 0.234 ^a^ |
| Female infertility duration (years) | 4.0 (4.0) | 4.5 (4.0) | 0.100 ^c^ |
| Female BMI | 22.1±3.3 | 22.0±3.3 | 0.803 ^b^ |
| Endometrial thickness (mm) | 9.1±1.8 | 9.2±2.2 | 0.708 ^b^ |
| Male age | 34.1±5.4 | 35.1±5.7 | 0.108 ^b^ |
| Male infertility type (n, %) |  |  |  |
| primary infertility | 559 (92.1%) | 48 (7.9%) |  |
| secondary infertility | 329 (92.2%) | 28 (7.8%) | 0.971 ^a^ |
| Male infertility duration (years) | 2.0 (4.0) | 2.0 (5.0) | 0.618 ^c^ |
| Male BMI | 24.3±3.5 | 24.5±3.3 | 0.614 ^b^ |
| Endometrial preparation protocol (n, %) |  |  |  |
| natural cycle | 184 (88.0%) | 25 (12.0%) |  |
| exogenous steroid replacement cycle | 704 (93.2%) | 51 (6.8%) | 0.013 ^a^ |
| Endometrial morphology (n, %) |  |  |  |
| A | 1 (100.0%) | 0 (0.0%) |  |
| A-B | 33 (91.7%) | 3 (8.3%) |  |
| B | 26 (81.3%) | 6 (18.8%) |  |
| B-C | 44 (93.6%) | 3 (6.4%) |  |
| C | 784 (92.5%) | 64 (7.5%) | 0.233 ^a^ |

^a^ Pearson χ² test. Values are number (percentage).

^b^ Two-sample t-test. Values are mean±SD.

^c^ Two-sample Mann–Whitney test. Values are median (IQR).
