# Supplementary material for: The impact of post-thaw culture time on clinical outcomes of embryos at different developmental stages
Source: Front Physiol. 2026 Oct 30;17:1929566. doi: 10.3389/fphys.2026.1929566 (PMC13759371; doi:10.3389/fphys.2026.1929566)
Supplement: Supplementary file 6 [file Table5.docx]

**Supplementary Table 5 Pooled multivariable logistic regression model for live‑birth outcome, including interaction between post‑thaw culture time and embryo developmental stage**

| Variable | *P*-value | OR (95% CI) |
| --- | --- | --- |
| Post-thaw culture time |  |  |
| < 4 h | Ref |  |
| ≥ 4 h | 0.006 ^a^ | 1.490(1.119, 1.985) |
| Female age | <0.001 ^b^ | 0.904(0.879, 0.930) |
| Female infertility type |  |  |
| primary infertility | Ref |  |
| secondary infertility | 0.337 ^a^ | 0.910(0.750, 1.103) |
| Female infertility duration | 0.031 ^b^ | 0.964(0.933, 0.997) |
| Female BMI | 0.100 ^b^ | 0.979(0.954, 1.004) |
| Endometrial thickness | <0.001 ^b^ | 1.116(1.069, 1.165) |
| Male age | 0.182 ^b^ | 0.983(0.959, 1.008) |
| Male infertility type |  |  |
| primary infertility | Ref |  |
| secondary infertility | 0.791 ^a^ | 0.974(0.802, 1.183) |
| Male infertility duration | 0.366 ^b^ | 0.986(0.956, 1.017) |
| Endometrial preparation protocol |  |  |
| natural cycle | Ref |  |
| exogenous steroid replacement cycle | <0.001 ^a^ | 1.583(1.269, 1.975) |
| Endometrial morphology |  |  |
| A | 0.877 ^a^ | 1.087(0.377, 3.131) |
| A-B | 0.340 ^a^ | 1.162(0.853, 1.584) |
| B | 0.930 ^a^ | 1.016(0.717, 1.439) |
| B-C | 0.995 ^a^ | 0.999(0.731, 1.366) |
| C | Ref |  |
| Embryo developmental stage |  |  |
| cleavage embryo | Ref |  |
| blastocyst | <0.001 ^a^ | 1.692(1.362, 2.102) |
| Number of transferred embryos |  |  |
| 1 | Ref |  |
| 2 | <0.001 ^a^ | 1.684(1.385, 2.048) |
| **Interaction: Post‑thaw culture time × Embryo stage** | **<0.001 ^a^** | **0.424(0.272, 0.663)** |

Multivariate analysis (binary logistic regression) was performed to adjust for variables that had a *P*-value < 0.10 in the univariate analysis, as well as those that showed significant between-group differences at baseline after stratification by post-thaw culture time.

^a^ *P*-value between each variable’s subgroups and reference group.

^b^ *P*-value of each variable’s overall effects after adjusting for the other variables.
