# Supplementary material for: The impact of post-thaw culture time on clinical outcomes of embryos at different developmental stages
Source: Front Physiol. 2026 Oct 30;17:1929566. doi: 10.3389/fphys.2026.1929566 (PMC13759371; doi:10.3389/fphys.2026.1929566)
Supplement: Supplementary file 7 [file Table6.docx]

**Supplementary Table 6 Univariate and multivariate analyses were conducted to assess the impact of post-thaw culture time on gestational age at birth, birth weight, and body length at birth in single** **cleavage-stage embryo transfer cycles**

|  | Gestational age at birth | | Birth weight | | Body length at birth | |
| --- | --- | --- | --- | --- | --- | --- |
|  | Univariate *P*-value ^a^ | Multivariate  *P*-value ^b^, B (95% CI) | Univariate *P*-value ^a^ | Multivariate  *P*-value ^b^, B (95% CI) | Univariate *P*-value ^a^ | Multivariate  *P*-value ^b^, B (95% CI) |
| Female age | 0.532 |  | 0.835 |  | 0.910 |  |
| Female infertility type |  |  |  |  |  |  |
| primary infertility | Ref |  | Ref |  | Ref |  |
| secondary infertility | 0.794 |  | 0.649 |  | 0.698 |  |
| Female infertility duration (years) | 0.720 |  | 0.045 | 0.027,  96.225(12.074, 180.376) | 0.102 |  |
| Female BMI | 0.252 |  | 0.479 |  | 0.864 |  |
| Endometrial thickness (mm) | 0.833 |  | 0.196 |  | 0.568 |  |
| Male age | 0.558 |  | 0.631 |  | 0.949 |  |
| Male infertility type |  |  |  |  |  |  |
| primary infertility | Ref |  | Ref |  | Ref |  |
| secondary infertility | 0.931 |  | 0.371 |  | 0.889 |  |
| Male infertility duration (years) | 0.421 |  | 0.843 |  | 0.617 |  |
| Male BMI | 0.723 |  | 0.986 |  | 0.960 |  |
| Post-thaw culture time | 0.004 | 0.003,  -0.332(-0.538, -0.127) | 0.428 | 0.250,  55.600(-42.452, 153.652) | 0.194 | 0.924,  -0.016(-0.368, 0.335) |
| Endometrial preparation protocol |  |  |  |  |  |  |
| natural cycle | Ref |  | Ref |  | Ref |  |
| exogenous steroid replacement cycle | 0.852 |  | 0.237 |  | 0.314 |  |
| Endometrial morphology |  |  |  |  |  |  |
| A ^c^ | / | / | / | / | / |  |
| A-B | 0.058 | 0.063,  2.218(-0.130, 4.566) | 0.157 |  | 0.218 |  |
| B | 0.521 | 0.996,  0.004(-1.459, 1.467) | 0.616 |  | 0.271 |  |
| B-C | 0.290 | 0.056,  1.259(-0.035, 2.554) | 0.193 |  | 0.140 |  |
| C | Ref |  | Ref |  | Ref |  |
| Gestational age at birth | / |  | 0.019 | 0.011,  226.939(58.832, 395.045) | 0.027 | 0.080,  0.541(-0.072, 1.154) |

Categorical variables were transformed into dummy variables in these analyses. The multivariate analysis was conducted to control for variables with *P*-values less than 0.1 in the univariate analysis. The post-thaw culture time was analyzed as a continuous variable.

^a^ The univariate analysis employed a simple linear regression model.

^b^ The multivariate analysis employed a multiple linear regression model.

^c^ No instances of type A endometrial morphology were observed in single cleavage-stage embryo transfer cycles.
