# Supplementary material for: The impact of post-thaw culture time on clinical outcomes of embryos at different developmental stages
Source: Front Physiol. 2026 Oct 30;17:1929566. doi: 10.3389/fphys.2026.1929566 (PMC13759371; doi:10.3389/fphys.2026.1929566)
Supplement: Supplementary file 8 [file Table7.docx]

**Supplementary Table 7 Univariate and multivariate analyses were conducted to assess the impact of post-thaw culture time on gestational age at birth, birth weight, and body length at birth in double** **cleavage-stage embryo transfer cycles**

|  | Gestational age at birth | | Birth weight | | Body length at birth | |
| --- | --- | --- | --- | --- | --- | --- |
|  | Univariate *P*-value ^a^ | Multivariate  *P*-value ^b^, B (95% CI) | Univariate *P*-value ^a^ | Multivariate  *P*-value ^b^, B (95% CI) | Univariate *P*-value ^a^ | Multivariate  *P*-value ^b^, B (95% CI) |
| Female age | 0.453 |  | 0.738 |  | 0.917 |  |
| Female infertility type |  |  |  |  |  |  |
| primary infertility | Ref |  | Ref |  | Ref |  |
| secondary infertility | 0.661 |  | 0.351 |  | 0.841 |  |
| Female infertility duration (years) | 0.057 | 0.099,  -0.053(-0.117, 0.010) | 0.055 | 0.320,  -8.437(-25.082, 8.209) | 0.162 |  |
| Female BMI | 0.420 |  | 0.961 |  | 0.471 |  |
| Endometrial thickness (mm) | 0.509 |  | 0.221 |  | 0.269 |  |
| Male age | 0.725 |  | 0.707 |  | 0.551 |  |
| Male infertility type |  |  |  |  |  |  |
| primary infertility | Ref |  | Ref |  | Ref |  |
| secondary infertility | 0.491 |  | 0.266 |  | 0.570 |  |
| Male infertility duration (years) | 0.993 |  | 0.284 |  | 0.619 |  |
| Male BMI | 0.444 |  | 0.557 |  | 0.970 |  |
| Post-thaw culture time | 0.642 | 0.593,  0.018(-0.047, 0.082) | 0.550 | 0.996,  0.045(-17.500, 17.590) | 0.476 | 0.621,  -0.016(-0.081, 0.048) |
| Endometrial preparation protocol |  |  |  |  |  |  |
| natural cycle | Ref |  | Ref |  | Ref |  |
| exogenous steroid replacement cycle | 0.334 |  | 0.786 |  | 0.225 |  |
| Endometrial morphology |  |  |  |  |  |  |
| A | 0.424 |  | 0.054 | 0.030,  -393.090(-748.925, -37.256) | 0.132 |  |
| A-B | 0.335 |  | 0.234 | 0.319,  57.174(-55.487, 169.835) | 0.506 |  |
| B | 0.605 |  | 0.285 | 0.332,  70.809(-72.566, 214.185) | 0.316 |  |
| B-C | 0.798 |  | 0.475 | 0.314,  66.751(-63.523, 197.026) | 0.692 |  |
| C | Ref |  | Ref | Ref | Ref |  |
| Live births count | <0.001 | <0.001,  -2.813(-3.141, -2.485) | <0.001 | <0.001,  -290.508(-400.381, -180.636) | <0.001 | 0.385,  -0.186(-0.605, 0.234) |
| Gestational age at birth | / | / | <0.001 | <0.001,  171.116(146.887, 195.345) | <0.001 | <0.001,  0.915(0.823, 1.008) |

Categorical variables were transformed into dummy variables in these analyses. The multivariate analysis was conducted to control for variables with *P*-values less than 0.1 in the univariate analysis. The post-thaw culture time was analyzed as a continuous variable.

^a^ The univariate analysis employed a simple linear regression model.

^b^ The multivariate analysis employed a multiple linear regression model.
