# Supplementary material for: The impact of post-thaw culture time on clinical outcomes of embryos at different developmental stages
Source: Front Physiol. 2026 Oct 30;17:1929566. doi: 10.3389/fphys.2026.1929566 (PMC13759371; doi:10.3389/fphys.2026.1929566)
Supplement: Supplementary file 9 [file Table8.docx]

**Supplementary Table 8 Univariate and multivariate analyses were conducted to assess the impact of post-thaw culture time on gestational age at birth, birth weight, and body length at birth in single** **blastocyst transfer cycles**

|  | Gestational age at birth | | Birth weight | | Body length at birth | |
| --- | --- | --- | --- | --- | --- | --- |
|  | Univariate *P*-value ^a^ | Multivariate  *P*-value ^b^, B (95% CI) | Univariate *P*-value ^a^ | Multivariate  *P*-value ^b^, B (95% CI) | Univariate *P*-value ^a^ | Multivariate  *P*-value ^b^, B (95% CI) |
| Female age | 0.001 | 0.261,  -0.046(-0.126, 0.034) | 0.083 | 0.229,  13.001(-8.211, 34.213) | 0.009 | 0.338,  0.035(-0.037, 0.107) |
| Female infertility type |  |  |  |  |  |  |
| primary infertility | Ref |  | Ref |  | Ref |  |
| secondary infertility | 0.195 |  | 0.837 |  | 0.837 |  |
| Female infertility duration (years) | 0.012 | 0.682,  -0.018(-0.106, 0.069) | 0.002 | 0.031,  -23.244(-44.296, -2.192) | 0.009 | 0.312,  -0.037(-0.109, 0.035) |
| Female BMI | 0.139 |  | 0.282 |  | 0.434 |  |
| Endometrial thickness (mm) | 0.656 |  | 0.536 |  | 0.911 |  |
| Male age | 0.001 | 0.461,  -0.026(-0.097, 0.044) | 0.037 | 0.668,  -4.072(-22.748, 14.603) | 0.002 | 0.217,  -0.040(-0.103, 0.023) |
| Male infertility type |  |  |  |  |  |  |
| primary infertility | Ref |  | Ref |  | Ref |  |
| secondary infertility | 0.186 |  | 0.623 |  | 0.978 |  |
| Male infertility duration (years) | 0.007 | 0.109,  -0.066(-0.146, 0.015) | 0.366 |  | 0.299 |  |
| Male BMI | 0.491 |  | 0.885 |  | 0.236 |  |
| Post-thaw culture time | 0.700 | 0.582,  -0.039(-0.179, 0.101) | 0.947 | 0.711,  6.965(-29.991, 43.921) | 0.971 | 0.705,  0.024(-0.101, 0.150) |
| Endometrial preparation protocol |  |  |  |  |  |  |
| natural cycle | Ref |  | Ref |  | Ref |  |
| exogenous steroid replacement cycle | 0.407 |  | 0.159 |  | 0.535 |  |
| Endometrial morphology |  |  |  |  |  |  |
| A | 0.227 |  | 0.218 | 0.438,  390.290 (-599.266, 1379.847) | 0.257 |  |
| A-B | 0.719 |  | 0.268 | 0.112,  173.785(-40.670, 388.241) | 0.906 |  |
| B | 0.466 |  | 0.075 | 0.129,  203.365(-59.480, 466.210) | 0.666 |  |
| B-C | 0.379 |  | 0.453 | 0.709,  -44.620(-279.835, 190.595) | 0.798 |  |
| C | Ref |  | Ref | Ref | Ref |  |
| Live births count | <0.001 | <0.001,  -2.610(-4.066, -1.155) | <0.001 | 0.156,  -280.138(-667.826, 107.549) | 0.001 | 0.642,  -0.313(-1.636, 1.010) |
| Gestational age at birth | / | / | <0.001 | <0.001,  174.460(144.984, 203.937) | <0.001 | <0.001,  0.796(0.695, 0.896) |

Categorical variables were transformed into dummy variables in these analyses. The multivariate analysis was conducted to control for variables with *P*-values less than 0.1 in the univariate analysis. The post-thaw culture time was analyzed as a continuous variable.

^a^ The univariate analysis employed a simple linear regression model.

^b^ The multivariate analysis employed a multiple linear regression model.
