# Supplementary material for: The impact of post-thaw culture time on clinical outcomes of embryos at different developmental stages
Source: Front Physiol. 2026 Oct 30;17:1929566. doi: 10.3389/fphys.2026.1929566 (PMC13759371; doi:10.3389/fphys.2026.1929566)
Supplement: Supplementary file 10 [file Table9.docx]

**Supplementary Table 9 Univariate and multivariate analyses were conducted to assess the impact of post-thaw culture time on gestational age at birth, birth weight, and body length at birth in double** **blastocyst transfer cycles**

|  | Gestational age at birth | | Birth weight | | Body length at birth | |
| --- | --- | --- | --- | --- | --- | --- |
|  | Univariate *P*-value ^a^ | Multivariate  *P*-value ^b^, B (95% CI) | Univariate *P*-value ^a^ | Multivariate  *P*-value ^b^, B (95% CI) | Univariate *P*-value ^a^ | Multivariate  *P*-value ^b^, B (95% CI) |
| Female age | 0.724 |  | 0.116 |  | 0.098 | 0.076,  0.057(-0.006, 0.119) |
| Female infertility type |  |  |  |  |  |  |
| primary infertility | Ref |  | Ref |  | Ref |  |
| secondary infertility | 0.729 |  | 0.708 |  | 0.934 |  |
| Female infertility duration (years) | 0.197 |  | 0.852 |  | 0.586 |  |
| Female BMI | 0.418 |  | 0.431 |  | 0.692 |  |
| Endometrial thickness (mm) | 0.786 |  | 0.768 |  | 0.484 |  |
| Male age | 0.476 |  | 0.306 |  | 0.378 |  |
| Male infertility type |  |  |  |  |  |  |
| primary infertility | Ref |  | Ref |  | Ref |  |
| secondary infertility | 0.648 |  | 0.638 |  | 0.432 |  |
| Male infertility duration (years) | 0.647 |  | 0.966 |  | 0.548 |  |
| Male BMI | 0.025 | 0.081,  0.042(-0.005, 0.089) | 0.025 | 0.514,  2.958(-5.945, 11.861) | 0.047 | 0.483,  0.025(-0.045, 0.095) |
| Post-thaw culture time | 0.413 | 0.715,  -0.026(-0.168, 0.115) | 0.081 | 0.173,  -18.689(-45.580, 8.202) | 0.988 | 0.483,  0.076(-0.136, 0.287) |
| Endometrial preparation protocol |  |  |  |  |  |  |
| natural cycle | Ref |  | Ref |  | Ref |  |
| exogenous steroid replacement cycle | 0.344 |  | 0.521 |  | 0.500 |  |
| Endometrial morphology |  |  |  |  |  |  |
| A | 0.856 |  | 0.984 | 0.702,  145.053(-598.453, 888.560) | 0.669 |  |
| A-B | 0.853 |  | 0.436 | 0.139,  121.711(-39.582, 28.003) | 0.603 |  |
| B | 0.676 |  | 0.524 | 0.161,  -116.561(-279.784, 46.662) | 0.894 |  |
| B-C | 0.920 |  | 0.070 | 0.002,  -227.390(-371.041, -83.740) | 0.867 |  |
| C | Ref |  | Ref | Ref | Ref |  |
| Live births count | <0.001 | <0.001,  -2.266(-02.620, -1.912) | <0.001 | <0.001,  -314.064(-390.418, -237.711) | <0.001 | 0.879,  -0.047(-0.655, 0.561) |
| Gestational age at birth | / | / | <0.001 | <0.001,  176.886(160.662, 193.110) | <0.001 | <0.001,  0.985(-0.856, 1.114) |

Categorical variables were transformed into dummy variables in these analyses. The multivariate analysis was conducted to control for variables with *P*-values less than 0.1 in the univariate analysis. The post-thaw culture time was analyzed as a continuous variable.

^a^ The univariate analysis employed a simple linear regression model.

^b^ The multivariate analysis employed a multiple linear regression model.
